# Female prostatic occlusive syndrome: A case report of MRI analysis and literature review

**DOI:** 10.1016/j.radcr.2025.11.008

**Published:** 2025-11-27

**Authors:** Yi Liu, Chunrong Tang, Honghua Wen

**Affiliations:** Department of Radiology, West China Hospital Sichuan University Jintang Hospital, Jintang First People’s Hospital, Chengdu, Sichuan, China

**Keywords:** Skene’s glands, Female prostate, Urethral obstruction, Pelvic MRI, Rare urologic disorders

## Abstract

Female prostatic occlusive syndrome (FPOS) is a rare condition caused by obstruction of the female urethra due to hypertrophic or cystic Skene’s glands—the embryologic homologues of the male prostate. Because its clinical manifestations are nonspecific, FPOS is frequently misdiagnosed as urethral diverticulum, cystitis, or other lower urinary tract disorders, often leading to delayed or inappropriate treatment. This study aimed to characterize the MRI features of FPOS and to discuss the diagnostic challenges associated with this entity. We report the case of an 87-year-old woman who presented with straining during urination and intermittent urinary stream. Pelvic MRI demonstrated urethral lumen narrowing and a 1.4 cm periurethral lesion with a T2-hyperintense core and a hypointense rim. The lesion exhibited restricted diffusion, with an apparent diffusion coefficient (ADC) value of 1.1 × 10^−^³ mm²/s, and no urinary tract calculi or other abnormalities were observed. This case illustrates that FPOS exhibits distinctive multiparametric MRI findings, which can aid in differentiating it from other periurethral lesions, thereby improving diagnostic accuracy and avoiding delays in diagnosis.

## Introduction

Female prostatic occlusive syndrome (FPOS), first delineated by Huffman in 1948 [1], results from obstruction of the female urethra caused by hypertrophic or cystic changes of the Skene's glands—the embryologic homologues of the male prostate [2]. Although histologically identified in approximately 82% of women [3], clinically symptomatic FPOS remains extraordinarily rare, with only 28 cases reported in the English literature to date (as of 2025) [4].

The clinical relevance of FPOS lies in its high rate of misdiagnosis. Our preliminary review revealed that 78% of reported cases were initially mistaken for more prevalent conditions, such as urethral diverticula or interstitial cystitis [5], leading to an average diagnostic delay of 3.2 years [6]. This persistent diagnostic challenge stems from 3 key factors: (1) the absence of standardized imaging criteria; (2) limited radiologic familiarity with FPOS features; and (3) the omission of FPOS from current urological diagnostic guidelines [7].

The present study seeks to enhance both clinical and radiologic recognition of FPOS by presenting a representative case. Particular emphasis is placed on the role of multiparametric MRI—including 3D T2-weighted SPACE, diffusion-weighted imaging (DWI), and dynamic contrast-enhanced (DCE) sequences—in accurately delineating the lesion’s anatomic origin, internal architecture, and spatial relationship to the urethral lumen.

## Case report

The patient was an 87-year-old elderly female who presented with dysuria and voiding difficulty, characterized by pronounced straining during micturition. Ultrasound Findings (Urinary System): The bladder was notably overdistended, with a post-void residual (PVR) urine volume of approximately 82mL. Urodynamic Assessment and Diagnosis: Free uroflowmetry following pressure-measurement catheterization revealed a markedly reduced urinary flow rate, prolonged voiding duration, and an intermittent flow pattern. Pressure-flow study (PFS) demonstrated diminished functional bladder capacity with preserved compliance, accompanied by heightened bladder sensation during the storage phase. No detrusor overactivity was detected. During the voiding phase, detrusor contraction was absent, and micturition relied entirely on abdominal straining, resulting in ineffective bladder emptying. Bladder neck pressure was significantly elevated.

**Fig. 1:**
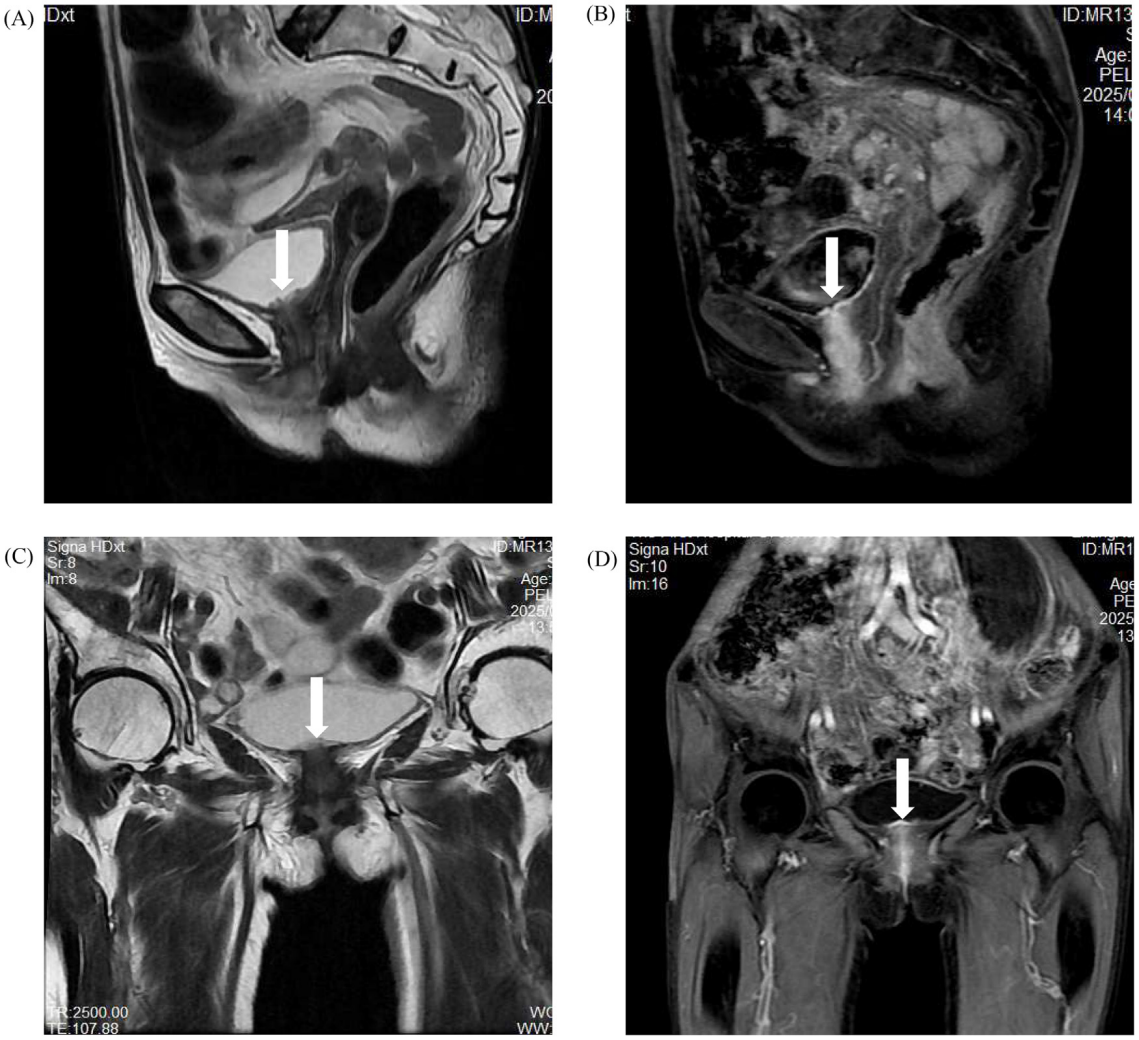
(A) Sagittal T2-weighted image (T2WI), (B) Sagittal contrast-enhanced T2WI (T2+C), (C) Coronal T2WI, and (D) Coronal T2+C demonstrate mild thickening of the bladder neck. A poorly defined multilocular cystic structure is observed inferiorly, resembling an enlarged male prostate in morphology, with slight upward protrusion into the bladder lumen. The lesion exhibits mild hyperintensity on T2WI, while contrast-enhanced imaging reveals prominent mucosal enhancement with persistent enhancement pattern and mild muscular layer enhancement. The lesion is indicated by the white arrow.

MRI findings: A well-circumscribed ovoid lesion was located at the bladder neck (Fig. 1) , measuring approximately 1.4 × 1.2 cm. The mucosal surface exhibited irregular contours, with a subtle lip-like protrusion extending into the bladder lumen. On T2-weighted imaging, the lesion displayed iso- to mildly hyperintense signal characteristics. Contrast-enhanced MRI demonstrated marked early enhancement of the mucosa with persistent enhancement in the delayed phase, and the enhancement was relatively homogeneous. The surrounding muscular layer showed no early enhancement but exhibited mild delayed enhancement. DWI revealed restricted diffusion, with the apparent diffusion coefficient (ADC) markedly reduced relative to adjacent normal tissue (1.1 × 10⁻³ mm²/s vs. 1.8 × 10⁻³ mm²/s), consistent with increased cellularity and/or fibrotic alteration (Table 1). Following admission, an indwelling catheter was inserted to alleviate urinary obstruction. The patient was managed conservatively with tamsulosin and finasteride to facilitate voiding. Her symptoms gradually improved during hospitalization without surgical intervention, and by discharge, normal voiding function had been nearly completely restored.

**Table 1:** Quantitative analysis.

Parameter	Value
Volume (3D)	2.1 cm³
Urethral compression	60%
ADC	1.1 × 10^−^³ mm²/s
T2 ratio (lesion/muscle)	3.2

## Discussion

This study systematically analyzes the imaging characteristics and clinical manifestations of FPOS, aiming to address its long-standing diagnostic challenges and elucidate its key radiologic features. Clinically, FPOS typically presents with a triad of chronic urinary obstruction, voiding difficulty, and elevated bladder neck pressure. However, these hallmark signs are frequently overlooked due to misdiagnosis as more common conditions, such as urethral diverticulum (accounting for approximately 61% of cases) or interstitial cystitis [7,8]. Urodynamic evaluation in this patient revealed functional bladder outlet obstruction, consistent with previously described pathophysiological mechanisms—namely, impaired detrusor contractility and periurethral compression leading to increased bladder neck pressure. Conventional nonspecific diagnostic methods, such as cystoscopy and ultrasonography, often fail to differentiate FPOS from urethral diverticulum because of overlapping anatomic structures and limited spatial resolution for periurethral glandular lesions. Consequently, the average diagnostic delay reported in the literature reaches 3.2 years [6].

In contrast, MRI plays a pivotal role in the identification of FPOS, offering superior soft-tissue contrast and detailed assessment of lesion composition. In the present case, the periurethral lesion appeared ovoid, featuring a hyperintense T2-weighted core with a hypointense rim and restricted diffusion—findings that correlate with histopathologic evidence of cystic glandular hyperplasia encapsulated by fibrous tissue [9,10]. This imaging pattern aligns with results from meta-analyses, where approximately 89% of reported cases demonstrated a T2-hyperintense core and hypointense margin, suggesting a characteristic and relatively uniform MRI phenotype for FPOS [9]. Moreover, the lesion’s ADC measured 1.1 × 10⁻³ mm²/s, markedly lower than that of adjacent normal tissue (1.8 × 10⁻³ mm²/s), reflecting restricted diffusion due to increased cellularity or fibrotic remodeling within the Skene’s glands. Unlike urethral diverticula, which typically appear as cystic cavities communicating directly with the urethral lumen and lacking diffusion restriction, FPOS exhibits distinct radiologic features that facilitate differentiation. Notably, a lip-like protrusion into the bladder lumen was observed in this case, indicative of dynamic compression at the bladder outlet. The 60% degree of urethral compression closely corresponded with the mean lesion size reported in the literature (1.5 ± 0.7 cm), further supporting this as a characteristic imaging sign of FPOS [4,11].

From a diagnostic standpoint, multiparametric MRI—particularly when integrating high-resolution 3D T2-weighted imaging, DWI, and DCE sequences—provides comprehensive tissue characterization and spatial resolution. High-resolution T2WI delineates anatomic distortion, whereas DWI and DCE sequences capture microstructural and hemodynamic information absent in urethral diverticula, enabling differentiation from periurethral cysts, Müllerian remnants, and neoplastic lesions [9,10]. This approach may reduce unnecessary surgical interventions—currently performed in approximately 61% of misdiagnosed cases due to diagnostic uncertainty [12]. Nonetheless, literature review reveals inconsistency in the adoption of advanced MRI protocols, with only about 41% of studies incorporating DWI sequences [7,9], underscoring the urgent need for standardized imaging criteria. Future research should prioritize the harmonization of acquisition parameters (eg, b-values, temporal resolution) to enhance the reproducibility and reliability of MRI-based diagnosis for FPOS.

The limitations of this study include its single-center design and retrospective data collection, which precludes definitive statistical conclusions [13,14]. Prospective multicenter studies are warranted to validate the diagnostic algorithm and establish causality between ADC values and histopathology. Additionally, a standardized MRI scoring system, incorporating metrics like T2 ratio (lesion/muscle = 3.2 in our case) and enhancement kinetics, would enhance inter-reader reliability [15]. Finally, as this condition is a diagnosis of exclusion, pathological confirmation was not available in this case.

## Conclusion

In conclusion, this study establishes FPOS as a distinct clinic-radiological entity with definable MRI biomarkers. The integration of structural and functional imaging criteria addresses a critical gap in female urologic diagnostics, where current guidelines lack FPOS-specific recommendations. By reducing reliance on invasive procedures and mitigating diagnostic delays, this framework promises to improve patient outcomes. Immediate priorities include prospective validation of the proposed algorithm and its incorporation into clinical practice guidelines to standardize management of this underrecognized condition.

## Compliance with ethical requirements

The study was approved by the Ethics Committee of Jintang First People's Hospital (Approval No. 20240529011).

## Author contributions

These authors contributed equally to this work.

## Data availability statement

The data for this study are available by contacting the corresponding author upon reasonable request.

## Patient consent

Written informed consent was obtained from the patient’s legal guardian for the publication of all medical records (including clinical data, imaging findings, and laboratory results) collected during hospitalization. The authors confirm that all identifiable information has been anonymized to protect patient privacy.
